# Handwriting Evaluation in School-Aged Children With Developmental Coordination Disorder: A Literature Review

**DOI:** 10.7759/cureus.35817

**Published:** 2023-03-06

**Authors:** Prishita Koul, Moh'd Irshad Qureshi, Rakesh K Kovela

**Affiliations:** 1 Department of Neurophysiotherapy, Ravi Nair Physiotherapy College, Datta Meghe Institute of Medical Sciences (Deemed to be University), Wardha, IND; 2 Department of Physiotherapy, Nitte Institute of Physiotherapy, Nitte (Deemed to be University), Mangalore, IND

**Keywords:** fine motor skills, children, developmental coordination disorder, evaluation, handwriting, scales

## Abstract

Despite widespread computer use, legible handwriting remains an important common life skill that requires more attention from schools and health professionals. Importantly, instructors and parents typically attribute the difficulties to laziness or a lack of effort, causing the youngster anger and disappointment. Handwriting issues are a public health concern in terms of both prevalence and consequences. Writing is a tough and diverse activity that requires cognitive, perceptual-motor, mental, and emotional talents. It is largely a motor process involving an effective level of motor organization that results in exact movement synchronization. Handwriting problems have been connected to developmental disorders such as developmental coordination disorder. For the affected youngsters, forming letters takes more work, and the kid may forget what he or she planned to write. School children’s primary handwriting issues include illegible writing, slow handwriting, and strained writing. Handwriting problems may lead to scholastic underachievement and low self-esteem. Because of this complication, some school-aged children develop handwriting difficulties, which cause psychological distress and learning impairments. In the treatment of children with bad handwriting, the therapeutic intervention has been demonstrated to be successful. We aimed to determine how efficient tools and scales are which assess handwriting in school-aged children having developmental coordination disorder. Keyword searches were conducted on Google Scholar and PubMed, yielding 45 results, eight of which met the inclusion requirements. We concluded that there are a lot of scales and tools to date but no scale focuses on the temporal and spatial parameters for handwriting evaluation.

## Introduction and background

A neurodevelopmental illness that is manifested by a significant lack of motor coordination, which creates an interface with academic competency, daily living chores, and recreational engagement, is known as developmental coordination disorder (DCD) [1,2]. The latest edition of the Diagnostic and Statistical Manual of Mental Disorders, Fifth Edition (DSM-5) published by the American Psychiatric Association states that a child with DCD has motor coordination that is below the norm for his or her chronological age, may have been labelled as "clumsy," and may have experienced delays in early motor milestones such as walking and crawling. Academic performance or daily living tasks are hampered by coordination issues with either gross or fine motor movements, or both. A medical illness or sickness has nothing to do with coordination issues (e.g., cerebral palsy, muscular dystrophy, visual impairment, or intellectual disability). If intellectual disability is present, the child's motor challenges go beyond what is predicted based on intelligence quotient (IQ) [3,4]. For freshers, in DCD research, there is a structural organization of behavioral and brain-based experimental information that can assist theorists to build connections between the hierarchy of explanation-brain, cognition, and motor action [5]. Based on this body of research, a (hybrid) multicomponent ecological systems theory model of performance and advancements in cognitive neuroscience will be developed. The three essential components of the paradigm are based on systems theory, and motor performance is judged by the interplay of person, task, and environmental constraints [6]. Individually, there is an interaction set of restrictions that skew our reaction capacities at every particular stage of development [7]. At the most fundamental biological level, genetic variables initiate maturational processes that shape physical system architecture such as brain networks, neuromuscular systems, and biomechanical linkage [8].

However, contextual circumstances are required to activate certain genotypic pictures, and phenotypic pictures also represent the outcome of "nature via nurture." These fundamental structures serve as the foundation for a wide range of internal activities, including cognition (e.g., executive functions), motor control processes (e.g., internal modeling), and motor learning (e.g., procedural learning), all of which can be influenced by physical activity over time. As a result, these processes and structures restrict an individual's (latent) mobility alternatives. External task limitations, such as the objectives, norms, and equipment connected with a certain activity, are outside of the body and unique to the work at hand [9]. Occupational therapy, physiotherapy, medication (e.g., methylphenidate), food (e.g., fatty acids combined with vitamin E supplementation), and education (teachers, parents, physical education) are all used as therapeutic interventions. Process-oriented methods concentrate on the components or bodily processes required to execute activities. Bottom-up techniques include sensory integration, kinesthetic training, perceptual training, and combinations. The idea underpinning DCD is that uplifting bodily functions including sensory integration, kinesthesia, muscle strength, core stability, visual-motor perception, and so on, leads to an enhanced skill execution [10]. Hand-eye coordination, combining a visually perceived item into physical output, graphic abilities, and even handwriting are all examples of fine motor skills. Weak fine motor control, a lack of muscle contraction coordination, and variations in impact rate and strength can all contribute significantly to obscured and incoherent handwriting; as a result, assessing fine motor control in handwriting movement is critical in the overall assessment of handwriting deficits [11].

Graph motility, whether drawing or writing, in the classroom, is indeed a vital fine motor skill. Handwriting of children with DCD has grown less legible and organized. Higher slowdown and acceleration peaks are analyzed by looping or scribbling processes [12]. Additionally, while duplicating literature, children with DCD write fewer letters than children without DCD. In comparison, they write faster and spend more time writing while holding their pen in place than kids without DCD. Children with DCD pause more frequently than their classmates without DCD, not less frequently. Three main theories have been put up to explain why DCD children's handwriting impairments exist, despite the fact that a variety of internal and environmental factors may contribute to the onset of dysgraphia. While the second hypothesis contends that people struggle to coordinate their motor efficiency, the first explanation contends that people struggle with muscular stiffness and the activation of muscular force. According to the third theory, developing one's motor abilities needs time. In other words, students struggle to convert from feedback to feedforward handwriting control, which compromises the consistency of a single motor pattern [12]. This study emphasizes extracting and exploring whether there are any scales and tools that assess the temporal and spatial parameters for the evaluation of handwriting.

Etiology and risk factors

Even though DCD is categorized as a continuous disorder, unlike Down syndrome, it does not usually have a single discrete emergence (which signifies a single gene mutation), and hence its border with other ongoing illnesses has been called into doubt. One of the major confounding variables of DCD is thought to be attention deficit hyperactivity disorder (ADHD), with a prevalence ranging from 35% to 50% of patients [13,14]. Another rather common comorbidity, like dyslexia, is specific language impairment, which has an estimated frequency of 32% in the DCD population [15]. Other co-occurring conditions include ocular anomalies (refractive errors, amblyopia, and strabismus), which have been linked to abnormalities in children with DCD's eye-hand coordination, ability to use visual cues, particularly to guide limb movements, and visual memory [16], hypermobility syndrome of the small joints, which has been linked to difficulties in children's handwriting tasks [17], and migraines without aura, which manifest with impairment in cognitive functions [18]. Preterm birth, whether defined as short gestational age at delivery or low birth weight, is the single risk factor consistently associated with DCD [19]. This is crucial because children with DCD who are in school tend to retreat from physical and social activities. Furthermore, children with DCD lose physical fitness over time and are more vulnerable to adopting a sedentary-related impairment such as cardiovascular compromises or malfunctioning and obesity [20]. Children and adolescents with poor motor skills, sometimes referred to as "clumsy," constitute a hidden minority who are at a risk of withdrawing from or being excluded from physical activity. Given the interdependence of activity, cardiorespiratory fitness, body fat, and coronary vascular disease, the discovery that motor incoordination reduces physical activity levels is relevant [21].

Pathophysiology

Children with DCD had a physiological connection between the sensorimotor network and the posterior cingulate cortex, precuneus, and posterior middle temporal gyrus that was disrupted, according to whole-brain resting-state imaging. This prevented them from using execution knowledge to its fullest potential and most likely hampered the acquisition of motor information [22]. Numerous research studies have advanced the idea that the basal ganglia, parietal lobe, and cerebellum may play a role in its development because this is a significant motor function and visuospatial deficiency. According to neuropsychological research, poor visuomotor cognition, low nonverbal abilities, and execution dysfunction impaired frontal problem-solving and praxis skills at the ideomotor, conceptual, visuoconstructive, and speech levels. There were also persistent emotional, behavioral, and social difficulties. The patient’s cognitive and affective symptoms strongly suggest that DCD is linked to the “cerebellar cognitive affective syndrome” (CCAS), which includes affective dysregulation, as well as executive, visuospatial, and linguistic impairments, and can accompany both acquired and developmental cerebellar disorders. Single-photon emission computed tomography (SPECT) functional neuroimaging revealed significant perfusion anomalies in supratentorial regions, which support skillful motor act execution (prefrontal lobe), behavioral and emotional control (prefrontal lobe), and visual information processing in individuals (occipital lobe). This study confirms earlier neuroanatomical findings that the cerebellum and the prefrontal, temporal, posterior parietal, and limbic cortices have strong neural connections. The lateral prefrontal cortex (PFC) communicates to the cerebellum through the dentate nucleus and thalamus, whereas the PFC connects to the cerebellum via pontine nuclei. Therefore, as a result, anatomoclinical configurations in patients appear to reveal that like CCAS, DCD may be caused by a disruption in the close functional complex interaction between the cerebellum and supratentorial brain regions required for such implementation of an organized motor function, affective regulation, and visuomotor processing. The dispersed cerebrocerebellar network, which supports movement, cognition, and emotion, may not have fully developed or be underdeveloped, which could explain the motor, cognitive, and emotional symptoms of DCD [23].

Features

Children with DCD struggle to tie their shoelaces, button their shirt buttons, open and close zippers, brush their teeth, and use dishes and utensils [11,24]. Since children with DCD frequently struggle with fine motor skills, especially handwriting, their schoolwork frequently does not represent their personal and professional growth [25]. There is, however, also proof of a more general academic impairment encompassing reading, working memory, and arithmetic abilities [26]. Although the disorder is initially diagnosed based on motor difficulties, it may progress to complicated psychosocial issues with challenges in peer relationships and social involvement [27], bullying [28], low self-esteem and a sense of competence [29], and psychological disorders that are internalized, such as anxiety and low mood [27]. In addition to secondary psychosocial effects, individuals with DCD are more likely to exhibit other developmental characteristics such as hyperactivity, difficulty interacting with others, and specific learning problems, particularly dyslexia [30].

Incidence and prevalence

DCD influences the child’s health and well-being, as well as involvement in everyday life, with a subsequent impact on the family. It affects 1.8% to 4.8% of children, with a boy-to-girl ratio of 1.9, according to some studies, but others believe this is a conservative estimate [31]. DCD is one of the most prevalent disorders afflicting school-aged children, accounting for 5-6% of all cases [1,24,31,32].

## Review

Method

Search Strategy

An open-date search method was used to search the literature. To find papers on DCD, we used the search phrases “developmental coordination disorder,” “fine motor,” and “hand movement.” To explore papers on handwriting evaluation in DCD, we used the phrases “developmental coordination disorder,” “handwriting,” “tools,” “scales,” “evaluation,” “parameters,” and other “fine motor skills.” This method was used on PubMed and Google Scholar. The search approach was revised.

Three reviewers examined the titles and abstracts acquired through search techniques (P.K., M.I.Q., R.K.K.). The full-text review was likewise performed by the same reviewers. One reviewer checked the reference list of research chosen for the review to find additional papers.

Inclusion/exclusion criteria for studies

Observational studies were among the requirements for eligibility. All English language full-text studies on humans were included in the study of school-aged participants aged 7 to 16. A scale or method for assessing handwriting in children with developmental coordination impairment must have been included in the research intervention. Between 2009 and 2022, the investigations were published in peer-reviewed journals.

Letters to the editor, conference abstracts or inadequate data, animal research, and a lack of original data were all exclusion factors. There were no limitations to the setting. Studies conducted before 2008 were not included.

Results

Study Characteristics

**Figure 1:**
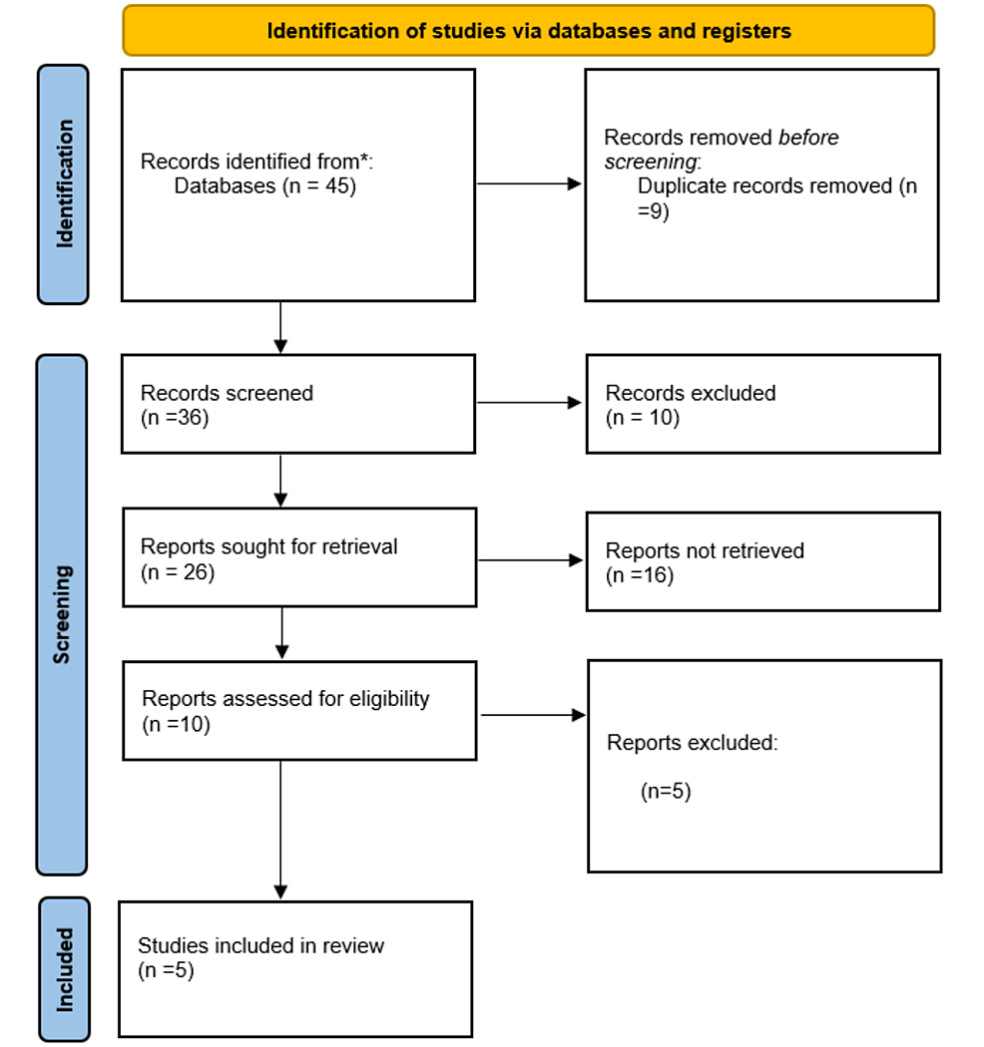
PRISMA flow diagram of the study selection process PRISMA, Preferred Reporting Items for Systematic Reviews and Meta-Analyses

A database search resulted in a total of 45 abstracts to examine. Following title/abstract screening, 10 papers were chosen for full-text examination. Following the full-text screening, five observational studies were included in our evaluation (Figure 1).

A total of 32 publications were found, of which five were subsequently selected in this review based on inclusion and exclusion criteria; a brief about these studies is given in Table 1.

**Table 1:** Summary of included studies DASH, Detailed Assessment of Speed of Handwriting; HLS, Handwriting Legibility Scale; ICC, intraclass correlation coefficient; PHAT, Persian Handwriting Assessment Tool; SOS, Systematische Opsporing van Schrijfmotorische problemen or “Systematic Screening of Handwriting Difficulties”

Study	Study Type	Study Sample	Method	Result	Conclusion
Barnett et al. (2009) [33]	Cross-sectional	546 school-going children	Writing the alphabet, 10 minutes of spontaneous scribbling, and a non-language assignment involving simply drawing crossing lines within concentric circles	The mean DASH score for copying, writing skills, and freestyle writing was 40, with a standard deviation of 10.	Their results indicate that the summative assessments on the first four activities, including a cumulative score of the speed of writing, may be used to offer evidence of slowness of handwriting and is a valid and reliable tool
Waelvelde et al. (2012) [34]	Cross-sectional	860 subjects	In 5 minutes, writing speed, letter fluency, letter connections, letter height and regularity, space between words, and word straightness are all evaluated	The reliability was high, and convergent validity was demonstrated by a correlation value of 0.70 between SOS and the "Concise Assessment Methods of Children Handwriting" test (Dutch version)	This exam differentiated between growing children, exceptional children, genders, and age groups. It has the potential to detect handwriting issues early on and prevent later complications as a result.
Rosenblum and Gafni-Lachter (2015) [35]	Cross-sectional	230 participants	Children were asked to write on lined paper with a pressure-sensitive tip that was the size of an A4 and mounted to the surface of a computer tablet for this study	The tool showed good internal consistency (α=0.77), reliability, and validity for the Hebrew version	Suitable for detecting handwriting deficiency in school-aged children and a variety of educational and therapeutic applications
Meimandi et al. (2018) [36]	Cross-sectional	452 healthy students	Copy and dictate 12 words on ruled paper, and the child’s handwriting will be evaluated for space, size, inclination, alignment, and letter formation	A low-to-moderate level of association was suggested by the criteria validity. Internal consistency (Cronbach's alpha: 0.72-0.99), test-retest reliability (ICC: 0.76-0.99), inter-rater reliability (ICC: 0.86-0.95), and inter-rater reliability (ICC: 0.86-0.95) were all excellent (ICC: 0.60-0.95)	According to the findings, the PHAT is a viable and accurate method for evaluating handwriting in primary school students
Fogel et al. (2022) [37]	Cross-sectional	148 children	Three of the four core DASH tasks were employed in this study (10-minute free-writing task, copying, and copying fast task). The HLS evaluates global readability, script effort, page layout, letter formation, and writing modifications	The findings revealed that letter output was the strongest determinant of total HLS score, although handwriting legibility varied significantly between tasks	For school-aged students, legibility of handwriting is necessary for copying and free-writing projects. As a task becomes more difficult and requires more sophisticated processing, text readability may suffer. During a study, it was found that the HLS is a sensitive method for determining handwriting readability

Barnett et al. conducted a research in which their objective for the Detailed Assessment of Speed of Handwriting (DASH) was to construct a set of tasks that accessed as many various components of handwriting speed as possible. Five tasks were completed by a stratified sample of 546 children who were between 9 and 16 years of age: (1) CopyBest; (2) Alphabet Writing; (3) CopyFast; (4) Graphic Speed; and (5) FreeWriting for 10 minutes, as well as an activity that didn't include language and entailed drawing crossing lines inside of concentric ring. The DASH Free Writing assignment is completed in 10 minutes, with the main raw score being the mean number of words per minute determined throughout the whole 10-minute time. The DASH focuses on handwriting speed rather than particular qualities of handwriting quality or letter formation. The evaluation has one limitation: the five tasks included in the DASH were deemed appropriate for children aged 9 years and above. The cognitive demands of the DASH activities may be disproportionate for most children younger than this age, rendering the findings difficult to evaluate. All five DASH activities are appropriate for kids up to the age of 16 years and are sensitive to developmental changes across the age range [33].

A research was conducted in 2012 in which they designed a brief, effective handwriting screening instrument to meet the demand. The SOS (Systematische Opsporing van Schrijfmotorische problemen or “Systematic Screening of Handwriting Difficulties”) is patterned on the brave handwriting kinder (BHK), but it may take less time to complete. This lets you check a child’s written text and afterward grade the overall BHK if more detailed information is needed to formulate an intervention plan. The six most discriminating factors explained 65% of the variation in pilot research (n = 128) and were therefore chosen from the 13 BHK criteria. To create the SOS test, they were rearranged and the scoring was streamlined. A total of 603 children aged 7 to 12 years old with an IQ of at least 70 but developmental issues were chosen from regular and special education schools. The writing speed of a child was measured by counting the written characters after he or she was asked to replicate a paragraph in 5 minutes. Seven items with scores ranging from 0 to 2 were used to assess writing quality. A total raw score was derived by summing these seven scores. On a transparent sheet supplied with the handbook, the components measuring letter height, regularity of letter height, and sentence perpendicular to the axis were measured. The study found that inter-rater and intra-rater reliability were excellent, while test-retest reliability was low. This test can be used to detect handwriting issues early on. This tool may help accomplish the objective of prompt intervention for children, preventing secondary issues such as scholastic underachievement and low self-esteem that are frequently connected with handwriting difficulties. The child’s capacity for sustained legible writing is not evaluated by the SOS. As a result, it is unknown if certain children’s legibility might diminish if they had to write for more than 5 minutes. The child’s writing pace varies according to the context, instruction, and whether he or she is copying, taking dictation, or free writing [34].

In 2015, researchers conducted a study to convert the previously recognized adults’ Handwriting Proficiency Screening Questionnaire (HPSQ) into a children’s self-report version (HPSQ-C) and assess its reliability and validity. A total of 230 Israeli youngsters aged 7 to 14 years from normal schools participated. The questionnaire’s contemporaneous and construct validity, internal consistency, and content validity were all assessed. There are three domains and 10 items in this questionnaire: performance time (items 3, 4, and 9), physical and emotional well-being (items 1, 2, and 10), and legibility (items 5-8). Each item is graded from 0 (never) to 4 (always); greater scores indicate poor performance. The HPSQ-C was determined to be adequate for assessing handwriting insufficiency in school-aged children, as well as for a range of academic and therapeutic tasks in this study. The HPSQ-C is most commonly used by occupational therapists [35].

In 2018, 452 healthy children aged 8 to 10 years were part of an observational study to assess several forms of Persian Handwriting Assessment Tool (PHAT) validity and reliability. Students were chosen using a random cluster sampling method. PHAT was created to evaluate handwriting legibility and speed. On a lined sheet of paper, the student should copy and describe 12 words for PHAT. Five readability taken into consideration by PHAT - separation (the distance between words and letters), breadth (appropriateness of word size), placement (word angle on the line), skewed (cumulative text angle on the line), and alphabet formation (correct ascending, descending, and rounding of letters) - are important factors in copying and dictation. This tool may be used independently and takes around 10 minutes to finish. The percentage of letters written every minute was computed as the number of letters divided by the number of seconds. Speed and orthographic mistakes were graded using a scale. The constituents of legibility (word structure, spacing, alignment, and text slant) were graded on a 5-point Likert scale (extremely poor to very good), with 5 being the highest score. The size was graded on a scale of 1 to 3, with 3 being the highest possible score. Finally, a participant’s score in both the copy and dictation domains was calculated using a mean of 12 words. The PHAT was discovered to be a viable and reliable way of evaluating handwriting in primary school-aged children. Students who are fluent in Persian, on the other hand, can profit from this application [36].

Fogel et al. explored handwriting readability across a range of writing activities, as well as the characteristics that predict overall handwriting legibility. The participants’ age in the research ranged from 9 to 14 years. Texts from the DASH free-writing exercise were combined to create the Handwriting Legibility Scale (HLS). Its goal was to evaluate five aspects of legibility: overall legibility (first-reading readability), total reading effort, page layout, letter formation, and writing modifications (attempts to rectify letters and words). Every component is assigned a score ranging from 1 (great performance) to 5, with a total score of legibility ranging from 5 to 25. Higher ratings imply that the material is difficult to read. After testing handwriting readability across multiple handwriting exercises, they concluded that HLS is a useful tool for occupational therapists who work in schools [37].

## Conclusions

In order to enable early intervention, which may prevent the secondary sequelae frequently associated with DCD, valid and reliable tools and scales for early identification of DCD are crucial. Researchers are arguing for an evidence-based, multi-professional, well-rounded assessment that includes motor assessments, parent/child questionnaires, and in-depth assessments of the impact on activities of daily living and positively contributes to family functioning and overall well-being. There appears to be a global interest in assessing younger populations. For evaluating handwriting in children with DCD, numerous scales and instruments are available. All of the aforementioned scales fall short when it comes to addressing the spatial and temporal aspects of handwriting. The scales are constrained and technologically advanced, which is not practical in the Indian setting, whether or not they are economically feasible. As a result, we believe that there is a considerable demand for a tool that analyses the spatial and temporal features of handwriting.
